# Secondary Metabolites and Their Bioactivities Produced by *Paecilomyces*

**DOI:** 10.3390/molecules25215077

**Published:** 2020-11-01

**Authors:** Ze-Bao Dai, Xin Wang, Guo-Hong Li

**Affiliations:** 1 State Key Laboratory for Conservation and Utilization of Bio-Resources in Yunnan, Yunnan University, Kunming 650091, China; yyg0517@163.com; 2 Key Laboratory for Microbial Resources of the Ministry of Education, Yunnan University, Kunming 650091, China

**Keywords:** *paecilomyces*, fungi, metabolites, bioactivities, structures

## Abstract

*Paecilomyces*, a common saprobic filamentous fungus, not only plays an important role in biological control, but also has applications in medicine, food, and environmental protection. In this paper, 223 secondary metabolites and their bioactivities from 13 known species and various unidentified strains of *Paecilomyces* are reviewed. Their structures can be described as polyketide, terpenoid, peptide, alkaloid, quinone, pyrone, sterol, and fatty acid. They have been demonstrated varying biological activities, including antimicrobial, antitumor, insecticidal, antiplasmodial, antimalarial, nematicidal, herbicidal, and enzyme-inhibiting. This review provides a comprehensive overview of secondary metabolites and their biological activities from strains of *Paecilomyces*.

## 1. Introduction

*Paecilomyces* is a common saprobic filamentous fungus. It is found in a wide range of habitats, including soils, forests, grassland, deserts, sediments, and even sewage sludge [1]. *Paecilomyces* belongs to the phylum Ascomycota, and the order Eurotiales, which has septate, branching hyphae, bearing long chains of conidia from the tips of conidiophores, and flask- to oval-shaped or subglobose phialide. Colonies of *Paecilomyces* are at first floccose and white, then become different colors. *Paecilomyces* strains do not harm to health in general and are in occasion opportunistic in humans and mammals.

Many species of *Paecilomyces* are important entomopathogenic fungi, which refer to a class that can infect or parasitize living host organisms and are an ecologically highly specialized group of micro-organisms. Entomopathogenic fungi are well known for their ability to produce various bioactive compounds during infection and proliferation in insects, and are considered as potential sources of novel bioactive compounds. The entomopathogenic fungi belonging to the genus *Paecilomyces* have been extensively studied as potential biological control agents against insects. Besides, *Paecilomyces* species have been used as Chinese traditional medicine to treat impotence, sedation, analgesia, backache, cancer, memory loss, and also as a tonic to nourish the lungs and kidneys [2]. Moreover, strains of *Paecilimyces* can survive in a wide range of temperatures and pH, which allows them to grow in a variety of substrates and makes them a rich source of biologically active natural products [3].

**Figure 1:**
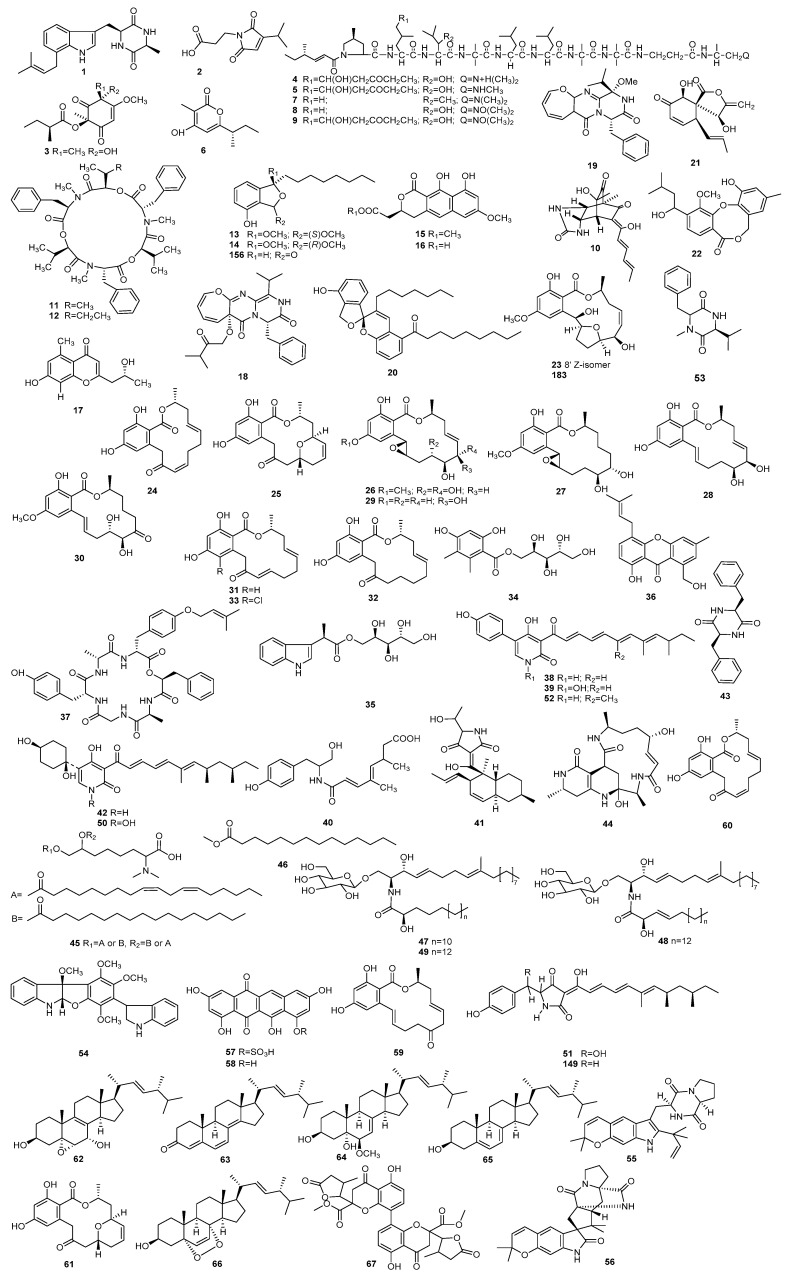
The structures of metabolites produced by *Paecilomyces* (**1**).

**Figure 2:**
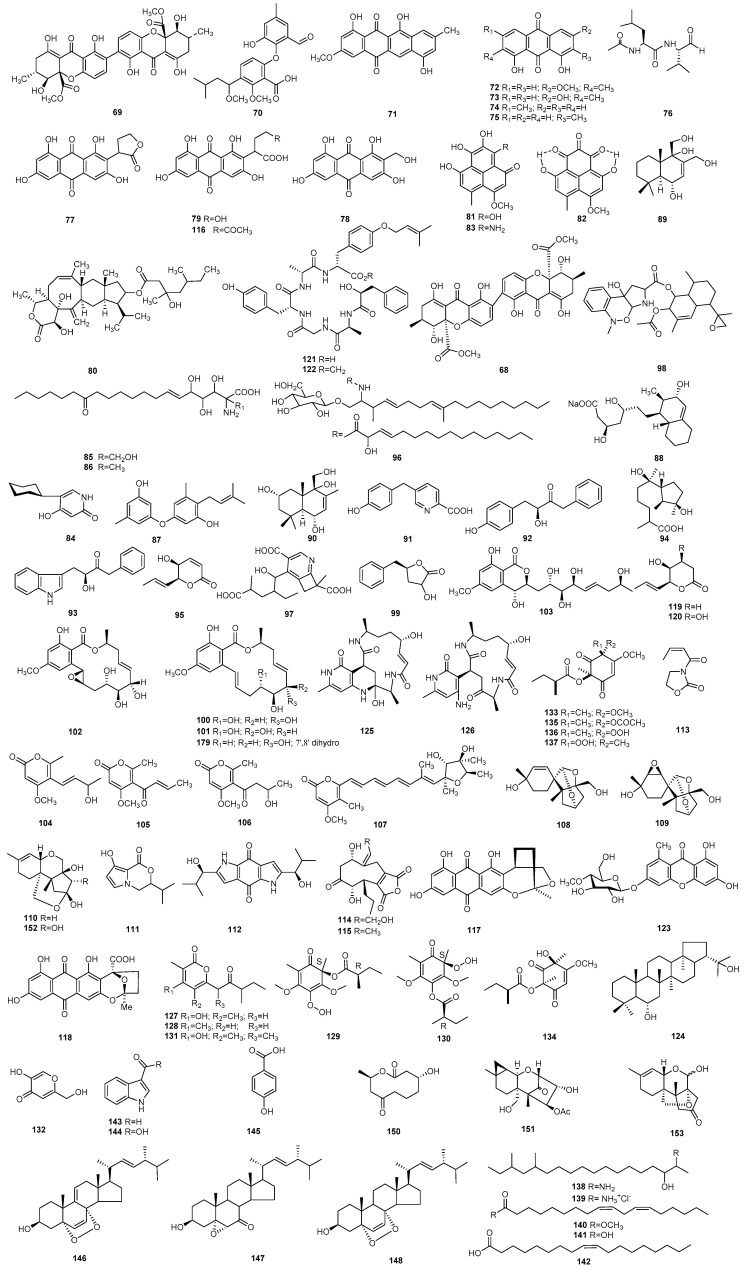
The structures of metabolites produced by *Paecilomyces* (**2**).

**Figure 3:**
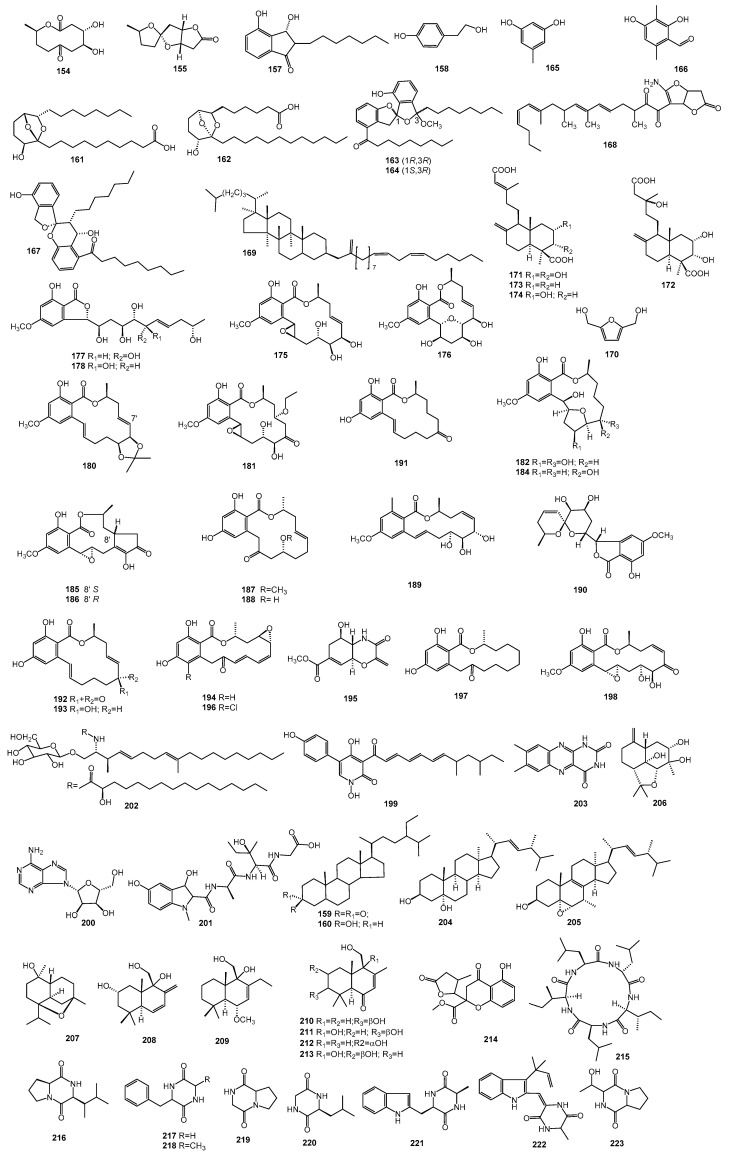
The structures of metabolites produced by *Paecilomyces* (**3**).

*Paecilomyces* is closely related to all aspects of human life and it plays an irreplaceable role in biological control, and has an important role in medicine and health. This review presents 223 secondary metabolites and their biological activities isolated from the 13 known species and various unidentified strains of *Paecilomyces*. The review covers reports from 1972 until the present. The structures of all compounds are summarized in Figures 1–3, and the active metabolites are concluded in Table 1.

## 2. Secondary Metabolites from *Paecilomyces*

### 2.1. Metabolites Derived from Paecilomyces with Antimicrobial Activity

Diketopiperazine terezine D (**1**) (Figure 1 and Table 1) was isolated from *P. cinnamomeus* BCC 9616, which was firstly found from the coprophilous fungus *Spwormiella teretispora*. It demonstrated activity against *Sordaria fimicola* (NRRL6459), causing a 50% reduction in the radial growth rate at 200 μg/disk [4,5]. A maleimide-bearing compound, farinomalein (**2**), was isolated from *P. farinosus* HF599, which showed potent activity against the plant pathogen *Phytophthora sojae* P6497 at 5 µg/disk [6]. α-Pyrone analogue phomaligol A (**3**) was identified from a strain of *P. lilacinus* derived from the marine sponge *Petrosia* sp. [7]. In addition, phomaligol A (**3**) was also obtained from the marine derived fungus *Aspergillus flavus* [8] and the blackleg fungus *Leptosphaeria maculans* [9], and exhibited antibacterial activity against *Staphylococcus aureus*, methicillin-resistant *S. aureus*, and multidrug-resistant *S. aureus* with minimum inhibitory concentration (MIC) values at 31.2–62.5 μg/mL [8]. The complex antibiotic, leucinostatin, was isolated from *P. lilacinus* A-267 [10], before being separated into leucinostatin A (**4**) and leucinostatin B (**5**). They demonstrated antimicrobial activity against bacteria and fungi [11,12]. The compound phomapyrone C (**6**) was identified from a strain of *P. lilacinus* [7] and *Aspergillus* sp. SCSIO 41024 isolated from deep-sea [13]. It had weak antibacterial activity against *Acinetobacter baumannii* with the MIC value of 250 μg/mL [13].

A series of peptidic antibiotics: leucinostatin A (**4**), D (**7**), H (**8**), and K (**9**), were identified from *P. marquandii* [14,15,16]. Leucinostatin D (**7**) showed biological activities against Gram-positive bacteria and several fungi, for example, *Bacillus subtilis* ICI, *Micrococcus luteus* ISS, *Streptococcus pneumonia*, *S. haemolyticus*, and *S. aureus* [15]. Leucinostatin H (**8**) and K (**9**) also exhibited activities against Gram-positive bacteria and fungi, but the antibacterial and the antimycotic activity reduce significantly upon *N*-oxidation [16]. A Diels-Alder product of sorbicillinoid (**10**) with a urea group was isolated from *P. marquandii*, a intertidal marine strain, which had antibacterial activity against *B. subtilis* ATCC 6633 and *E. coli* ATCC 25922 [17].

Cyclodepsipeptides beauvericin (**11**) and beauvericin A (**12**) were isolated from *P. tenuipes* BCC 1614. The two compounds displayed antimicrobial activities [18]. Beauvericin (**11**) can also be obtained from several fungi, including *Beauveria bassiana*, *Polyporus sulphureus*, and *Fusarium* spp. [19,20,21,22].

Two polyketides paecilocin B (**13**) and C (**14**) were identified from *P. variotii* derived from the jellyfish *Nemopilema nomurai*, which showed moderate antibacterial activity against *S. aureus* SG 511 and MRSA 3089 with MIC values ranging from 5 to 40 μg/mL [23]. Two metabolites, semi-viriditoxin (**15**) and semi-viriditoxic acid (**16**), were produced by a strain of *P. variotii*, isolated from the larvae of *Dendroctonus ponderosa*, and the two metabolites (**15**, **16**) showed weak antibacterial activity against a number of bacteria [24]. One chromone, lawsozaheer (**17**) was isolated from the broth of *Paecilomyces variotii*. It demonstrated highly selective activity against *S. aureus* (NCTC 6571) with 84.26% inhibition at 150 μg/mL [25]. Two oxepine-containing diketopiperazine-type alkaloids, varioloid A (**18**) and B (**19**), were identified from the marine alga-derived *P. variotii* EN-291, exhibiting potent activity against the plant pathogenic fungus *Fusarium graminearum* with MIC values of 8 and 4 mg/mL, respectively [26]. A benzannulated spiroketal derivative, paeciloketal A (**20**), was obtained from *P. variotii* J08NF-1, a jellyfish-derived strain, which showed antibacterial activity with a MIC value of 40 μg/mL against the marine pathogen *Vibrio ichthyoenteri* [27].

A metabolite paecilospirone (**21**), was reported from *Paecilomyces* sp., with a MIC value of 5 μg/mL against *B. subtilis* at 25 °C; however, at 37 °C, it did not show any antimicrobial activity [28]. Paeciloxocin A (**22**) was isolated from *Paecilomyces* sp., and it inhibited the growth of *Curvularia lunata* and *Candida albicans* ATCC 10231 with inhibition zones of 12 and 10 mm, respectively [29]. Paecilomycin M (**23**), monocillin VI (**24**) and VII (**25**), aigilomycin B–D (**26**–**28**), 1′,2′-epoxy aigialomycin D (**29**), LL-Z1640-1 (**30**), monocillin II (**31**), monocillin IV (**32**), and monorden D (**33**) were produced by *Paecilomyces* sp. SC0924. Compounds **23**–**30** exhibited weak antifungal activity against *Peronophythora litchi* [30,31,32]. Metabolites **31**–**33** can be separated from *Pochonia chlamydosporia* and demonstrated modest activity against *Xanthomonas campestris*, with a MIC value of 25.6 μg/mL [33]. Aigialomycin-type compound was also reported to be derived from *Aigialus parvus* [34,35]. LL-Z1640-1 (**30**) was firstly isolated from an unidentified fungus [36] and was also obtained from the gorgonian derived fungus *Cochliobolus lunatus* [37].

The antimicrobial and cytotoxic polyketide paeciloside A (**34**) and the compound acremoauxin A (**35**) were identified from a strain of *Paecilomyces* sp. CAFT156 [38]. Compounds **34** and **35** displayed inhibitory effects on two bacteria *B. subtilis* and *S. aureus* at 40 μg/disk [38]. A metabolite, paeciloxanthone (**36**), was obtained from *Paecilomyces* sp. (tree 1–7), a strain isolated from an estuarine mangrove from the Taiwan Strait. Metabolite **36** is active against *C. lunata*, *E. coli*., and *C. albicans* at 40 μg/disk, producing inhibitory zones of 6, 12, and 10 mm, respectively [39].

### 2.2. Cytotoxic Metabolites Derived from Paecilomyces

An antitumor cyclohexadepsipeptide, paecilodepsipeptide A (**37**), was derived from *P. cinnamomeus* BCC 9616. Paecilodepsipeptide A (**37**) exhibits cytotoxicity against cancer cell lines, KB and BC, with IC_50_ (the half maximal inhibitory concentration) values of 5.9 and 6.6 µM, respectively [40]. 

Farinosone A–C (**38**–**40**), three neurotrophic alkaloidal metabolites produced by *P. farinosus* RCEF 0101. Farinosone A (**38**) and C (**40**) can induce neurite outgrowth in the PC-12 cell line at concentrations of 50 µM, while farinosone B is inactive [41]. A tetramic acid derivative, paecilosetin (**41**), along with farinosone B (**39**), was isolated from *P. farinosus*. The two metabolites showed activity against the P388 cell line with IC_50_ values of 3.1 and 1.1 µg/mL, respectively [42]. A pyridone alkaloid, (+)-*N*-deoxymilitarinone A (**42**), was obtained from a strain of *P. farinosus* RCEF 0097. Compound **42** induced neurite sprouting in the PC-12 cell line when tested at concentrations of 33 and 100 µM and a cytotoxic effect was observed in human neurons (IMR-32) at 100 µM [43]. The metabolite (3S,6S)-3,6-dibenzylpiperazine-2,5-dione (**43**) was isolated from a culture extract of marine-derived *P. formous* 17D47-2; it showed selective cytotoxic activity in human pancreatic carcinoma PANC-1 cells adapted to glucose-starved conditions, with an IC_50_ value of 28 µM, whereas no effect against PANC-1 cells under general culture conditions up to 1000 µM [44].

A novel macrocyclic, tetralactams gunnilactam A (**44**), isolated from *P. gunnii*, exhibited cytotoxic activity against human prostate cancer C42B cells with an IC_50_ value of 5.4 μM [45].

A series of metaboilites, including 1,2-dilinolylglycero-*O*-4′-(*N*,*N*,*N*-trimethyl) homoserine (**45**), methyl myristate (**46**) [46] and cerebroside B–D (**47**–**49**) [47], were isolated from marine-derived *P. lilacinus* ZBY-1. The metabolites 45 and 46 inhibited the human cancer K562, MCF-7, HL-60, and BGC-823 cells lines with the IC_50_ values ranging from 1.12 to 8.63 μmol/L [46]. The compounds cerebroside B–D (**47**–**49**) inhibited K562, MCF-7, HL-60, and BGC-823 cells with IC_50_ values ranging from 9.5 to 59.6 mg/L [47]. Leucinostatin A (**4**) and B (**5**), derived from *P. lilacinus* A-267, as well as having antimicrobial activity, also showed antitumor activity and an uncoupling effect on rat liver mitochondrial function [11,12].

Three novel pyridone alkaloids, militarinone A (**50**), B (**51**), and D (**52**), were isolated from the mycelium of *P. militaris* [48,49]. Militarinone A (**50**) had a pronounced neurotrophic effect in the PC-12 cells at concentration of 10 µM [48]. Militarinone D (**52**) showed significant cytotoxicity against PC-12 cells with 74.0% and 30.7% at concentrations of 100 and 33 µM, respectively, and militarinone B (**51**) was weakly cytotoxic at 100 µM (16.8%) [49]. In addition, militarinone B (**51**) and D (**52**) can also be obtained from a strain of *P. farinosus* RCEF 0097 [43].

A peptidic antibiotic, leucinostatin D (**7**), was obtained from *P. marquandii*. The phytotoxicity test on tomato cuttings proved positive at 2 μg/mL, and *in vitro* cytotoxic activity assays showed that it inhibited HeLa, KB, and P388/S with ID_50_ values of 850, 0.95, and l.00 ng/mL [15].

The novel metabolite (3*S*)-6-phenethyl-3-isopropyl-1-methyl-2,5-diketopiperazine (**53**) was obtained from *P. tenuipes* and showed cytotoxicity against 22RV1 and DU-145 prostate cancer cells with inhibition rates of 37.8% and 38.6% at 5 µm/L [50]. Cyclodepsipeptide beauvericin (**11**) and beauvericin A (**12**) derived from *P. tenuipes* BCC 1614 also showed cytotoxic activity [18].

A series of compounds, including a indolyl-6,10b-dihydro-5a*H*-[1]benzofuro[2,3-*b*]indole derivative (**54**), a diketopiperazine-type alkaloid varioloid B (**19**), and two prenylated indole alkaloids dihydrocarneamide A (**55**), and *iso*-notoamide B (**56**), were identified from the marine alga-derived *P. variotii* EN-291 [26,51,52]. Compounds **19** and **54** exhibited cytotoxicity against A549, HCT116, and HepG2 cell lines, with IC_50_ values from 2.6 to 8.2 µg/mL [51]. Dihydrocarneamide A (**55**) and *iso*-notoamide B (**56**) showed cytotoxic activities against NCI-H460 with IC_50_ values of 69.3 and 55.9 mmol/L, respectively [52].

Three metabolites, UCE1022 (**57**), saintopin (**58**), and paeciloxocin A (**22**), were identified from an unidentified species of *Paecilomyces*. UCE1022 (**57**) displayed *in vitro* cytotoxic activity against HeLa S3 at IC_50_ 6.1 μM [53]. Saintopin (**58**) shows *in vitro* cytotoxic activity against HeLa S3 at IC_50_ 0.35 μg/mL, and further demonstrated *in vivo* antitumor activity against murine leukemia P388 (ip) [54]. Paeciloxocin A (**22**) exhibited significant cytotoxicity against hepG2 with an IC_50_ value of 1 μg/mL [29]. A β-resorcylic acid lactone, paecilomycin P (**59**), and two radicicol-type metabolites, monocillin VI and VII (**60**, **61**) were produced by a strain of *Paecilomyces* sp. SC0924 [32]. The three compounds (**59**–**61**) exhibited cytotoxicity against MCF-7, A549, and HeLa cells [32]. The metabolites paeciloside A (**34**) and acremoauxin A (**35**) were identified from *Paecilomyces* sp. CAFT156. The two compounds displayed moderate cytotoxicity towards *Artemia salina* [38]. The cytotoxic ergosterols, including 5α,6α-epoxy-(22*E*,24*R*)-ergosta-8,22-diene-3β,7α-diol (**62**), ergosta-4,6,8(14),22-tetraene-3-one (**63**), 3β,5α-dihydroxy-6β-methoxyer-gosta-7,22-diene (**64**), ergosterol (**65**), and ergosterol endoperoxide (**66**), were produced by *Paecilomyces* sp. J300 [55]. These compounds showed moderate cytotoxicity against A549, SK-OV-3, SK-MEL-2, XF498 (CNS), and HCT15 cells [55]. A sequence of metabolites, including paeciloxanthone (**36**) [39], paecilin A (**67**), secalonic acid D (**68**), secalonic acid A (**69**), tenellic acid A (**70**), and five anthraquinone derivates, tetracenomycin D (**71**), physcioin (**72**), emodin (**73**), chrysophanol (**74**), 1,4-dihydroxy-2-methy anthraquinone (**75**) were obtained from *Paecilomyces* sp. (tree 1–7) [39,56,57,58]. Paeciloxanthone (**36**) exhibited *in vitro* cytotoxicity against hepG2 with an IC_50_ value of 1.08 μg/mL [39]. Paecilin A (**67**) showed inhibiting activity against KB and KBv cells with IC_50_ values of 40 and 50 nmol/mL, respectively [56]. Secalonic acid D (**68**) showed cytotoxicity towards KB cells with an value of IC_50_ < 1 µg/mL and inhibited human topoisomerase I with an IC_50_ value of 0.16 µmol/mL [57]. Secalonic acid A (**69**) (Figure 2) and tenellic acid A (**70**) inhibited the growth of the human hepatoma cell line HepG2, with IC_50_ values of 62.1 and 2.0 μg/mL, respectively [58]. Compounds **71**–**75** showed anticancer activity against KB and KBv, with IC_50_ values of 11, 20, 8, 15, and 18 μmg/mL and 17, 30, 10, 20, and 25 μmg/mL, respectively [59].

### 2.3. Metabolites with Enzyme Inhibitory Activity from Paecilomyces

Paecilopeptin (**76**) is a novel cathepsin S inhibitor produced by *P. carneus*, which inhibits human cathepsin S *in vitro* with an IC_50_ value of 2.1 nM [60]. A series of inhibitors of the protein tyrosine kinases paeciloquinone A (**77**), C (**78**), and D (**79**) were obtained from *P. carneus* P-177 [61,62]. Paeciloquinone A (**77**) and C (**78**) are potent and selective inhibitors of the v-abl protein tyrosine kinase with an IC_50_ value of 0.4 μM [61]. Paeciloquinone D (**79**) is a protein kinase C inhibitor with an IC_50_ value around 6 μM [63].

Two metabolites, sester-terpenoid YW3548 (**80**) and a cyclic peptide paecilodepsipeptide A (**37**), were isolated from endophytic *P. formosus* LHL10 [64]. The two compounds exhibited remarkable inhibitory rates against α-glucosidase and urease, with IC_50_ values of 61.80 ± 5.7 and 75.68 ± 6.2, and 74.25 ± 4.3 and 190.5 ± 10.31 µg/g, respectively [64], which were also obtained from *P. cinnamomeus* [40]. Paecilomycone A–C (**81**–**83**) were identified from *P. gunnii* with IC_50_ values of 0.11, 0.17, and 0.14 mM on Tyrosinase, respectively [65]. A pyridone alkaloid, paecilomide (**84**), derived from *P. lilacinus*, demonstrated an acetylcholinesterase inhibition of 57.5 ± 5.50% [66].

Sphingofungins E (**85**) and F (**86**) are novel structures in the sphingofungin family which can inhibit serinepalmitoyl transferase at nanomolar levels; the estimated IC_50_ values were 7.2 and 57 nM, respectively, which were obtained from a strain of *P. variotii* ATCC 74097 [67]. A oxybis cresol, verticilatin (**87**), was identified from cultures of *P. verticillatus*. Verticilatin (**87**) exhibited significant inhibitory activity against CDC25B, cathepsin B, MEG2, and SHP2 enzyme, with IC_50_ values of 11.5, 3.5, 7.8, and 15 μg/mL, respectively [68]. A metabolite of the compactin family, 3α-hydroxy-3,5-dihydro ML-236C (**88**), was isolated from *P. viridis* L-68, and the *in vitro* activity of HMG-CoA reductase was inhibited by approximately 50% by this compound **88** [69].

The cadinane-type sesquiterpenoid analogs, 12-hydroxyalbrassitriol (**89**) and 2-hydroxyalbrassitriol (**90**), were obtained from the endophytic fungus *Paecilomyces* sp. TE-540. The two compounds showed moderate activities against acetylcholinesterase (AChE), with IC_50_ values of 43.02 ± 6.01 and 35.97 ± 2.12 μM, respectively [70]. Phenopicolinic acid (**91**), a potent inhibitor of dopamine β-hydroxylase, was found in culture filtrates of *Paecilomyces* sp. AF2562. The LD_50_ (median lethal dose or concentration) of phenopicolinic acid (**91**) for mice was about 350 mg/kg through intraperitoneal injection [71].

Two novel protein farnesyltransferase (PFTase) inhibitors, kurasoin A (**92**) and B (**93**), were derived from the cultured broth of *Paecilomyces* sp. FO-3684 [72]. The two metabolites inhibited PFTase in a dose-dependent, with IC_50_ values of 59.0 and 58.7 μM, respectively [72]. The metabolites paeciloxanthone (**36**) and secalonic acid D (**68**) were isolated from *Paecilomyces* sp. (tree 1–7). Paeciloxanthone (**36**) exhibited *in vitro* AChE inhibition with an IC_50_ value of 2.25 μg/mL [39], and secalonic acid D (**68**) inhibited human topoisomerase I with an IC_50_ value of 0.16 µmol/mL [57].

### 2.4. Insecticidal, Nematicidal, Antiplasmodial, and Antimalarial Metabolites Derived from Paecilomyces

Catenioblin C (**94**) and phomalactone (**95**) were identified from *P. cateniobliquus* YMF1.01799 [73]. The polyketide-derived phomalactone (**95**) had a significant inhibitory effect on the growth of the cotton bollworm *Helicoverpa armigera*, while the terpenoid derived metabolite catenioblin C (**94**) promoted the growth of the larvae [73]. Beauvericin (**11**) and beauvericin A (**12**) with diversiform bioactivities obtained from *P. tenuipes* BCC 1614, also demonstrated promising insecticidal activity [18]. The metabolite cerebrosides A (**96**) was isolated from marine-derived *P. lilacinus* ZBY-1, and its nematicidal activity against *Bursaphelenchus xylophilus* was investigated. The result showed that the average mortality of *B. xylophilus* treated with cerebroside A (**96**) at the mass concentrations of 1000, 100, and 10 μg/mL were 100%, 100%, and 11.1%, respectively [74].

A nematicidal metabolite 4-(4′-carboxy-2′-ethyl-hydroxypentyl)-5,6-dihydro-6-methyl- cyclobut[b]pyridine-3,6-dicarboxylic acid (**97**), was produced by *Paecilomyces* sp.YMF1.01761. Within 24 h, the LD_50_ value was 50.86 mg/L against *Panagrellus redivivus*, 47.1 mg/L against *Meloidogyne incognita*, and 167.7 mg/L against *B. xylophilus* [75]. Paeciloxazine (**98**) was isolated from *Paecilomyces* sp. BAUA3058, demonstrating moderate nematicidal activity agains*t Rhabditis pseudoelongata* and weak activity against some insects [76].

The metabolite paecilodepsipeptide A (**37**) was obtained from *P. cinnamomeus* BCC 9616. It possesses three D-amino acid residues and can act against the malarial parasite *Plasmodium falciparum* K1, with an IC_50_ value of 4.9 µM [4,40]. The compound harzialactone A (**99**) was isolated from the marine-derived fungus *Paecilomyces* sp. 7A22. It exhibited significant activity against *Leishmania amazonensis* with an IC_50_ value of 5.25 mg/mL and a moderate activity against intracellular amastigotes with an IC_50_ value of 18.18 mg/mL [77].

Two novel β-resorcylic acid lactones, paecilomycin E, F (**100**, **101**), along with aigilomycin B (**102**) and aigialomycin F (**103**) were isolated from a strain of *Paecilomyces* sp. SC0924 [78]. Paecilomycin E (**100**) and aigialomycin F (**103**) exhibited antiplasmodial activity against the *Plasmodium falciparum* line 3D7 with IC_50_ values of 20.0 and 10.9 nM, respectively, and paecilomycin E, F (**100**, **101**) and aigilomycin B (**102**) showed moderate activity against the *P. falciparum* line Dd2 [78]. Four metabolites containing pyrenocine I (**104**), pyrenocine A, B (**105**, **106**) and citreoviridin (**107**) were produced by *Paecilomyces* sp. FKI-3573 [79]. These compounds exhibit *in vitro* antitrypanosomal activity, and pyrenocine A (**105**) showed the most potent activity with an IC_50_ value of 0.12 mg/mL [79].

### 2.5. Other Active Metabolites Derived from Paecilomyces

The metabolites spirotenuipesine A, B (**108**, **109**) and paecilomycine A (**110**) were obtained from *P. tenuipes*. The three compounds showed potent activity in neurotrophic factor biosynthesis in glial cells [80,81]. A pyrrolooxazine, formoxazine (**111**), a dipyrroloquinone derivative, terreusinone (**112**), and a 2-oxazolidinone analogue, 3-[(2Z)-1-oxo-2-buten-1-yl]oxazolidin-2-one (**113**) were isolated from the marine-derived *P. formosus* [82]. The compounds **111** and **113** displayed potent radical-scavenging activity against DPPH, with IC_50_ values of 0.1 and 10 μM [82]. Terreusinone (**112**) exhibited a UV-A absorbing activity with an ED_50_ value of 70 µg/mL [83]. Phytotoxin 14-hydroxycornexistin (**114**), a member of the nonadride family, was obtained from *P. variotii*, exhibiting a potent activity against broadleaf weeds and a selectivity to corn [84]. A herbicidal antibiotic, cornexistin (**115)**, was isolated from *P. variotii* SANK 21086, which shows non-selective, broad spectrum herbicidal activity against annual plants including mono- and dicotyledonous weeds and may be useful for postemergence weed control with selective protection of corn [85,86]. In addition, cornexistin (**115**) was also isolated from *P. tenuipes* [25].

### 2.6. Metabolites with Unknown Activity Derived from Paecilomyces

The metabolites paeciloquinone B (**116**) and paeciloquinone E, F (**117**, **118**) were obtained from *P. carneus* P-177 [61,62]. Two compounds, catenioblin A, B (**119**, **120**) were identified from *P. cateniobliquus* YMF1.01799 for the first time [73]. The metabolites paecilodepsipeptide B and C (**121**, **122**), a xanthone glycoside, norlichexanthone-6-*O*-(4-*O*-methylglucopyranoside) (**123**), and hopane triterpene zeorin (**124**) were produced by *P. cinnamomeus* BCC 9616 [4,40]. Two novel macrocyclic tetralactams, gunnilactam B, C (**125**, **126**) were produced by *P. gunnii* [45].

A series of compounds, including two α-pyrones, paecilopyrone A, B (**127**, **128**); two cyclohexenones, phomaligol B, C (**129**, **130**); and the analogues, phomapyrone B (**131**) and C (**6**), kojic acid (**132**), phomaligol A (**3**), methylphomaligol A (**133**), phomaligol A_1_ (**134**), acetylphomaligol A (**135**), phomaligol A hydroperoxide (**136**), and phomaligol A_1_ hydroperoxide (**137**), were identified from a strain of *P. lilacinus* derived from the marine sponge *Petrosia* sp. The compounds kojic acid (**132**), phomaligol A (**3**), and methylphomaligol A (**133**) were evaluated for their cytotoxicity against a small panel of human solid tumor cell lines and were found to be inactive up to a concentration of 30 µg/mL [7]. In addition, phomaligol A_1_ (**134**) can also be obtained from the blackleg fungus *L. maculans* [9]. Eleven metabolites, including paecilaminol (**138**), paecilaminol hydrochlorate (**139**), methyl linoleate (**140**), linoleate (**141**), oleinic acid (**142**), indole-3-carboxaldehyde (**143**), indolyl-3-carboxylic acid (**144**), 4-hydroxybenzoic acid (**145**), 9(11)-dehydroergosterol peroxide (**146**), (22*E*,24*R*)-5α,6α-epoxy-3β-hydroxyergosta-22-ene-7-one (**147**), and ergosterol peroxiden (**148**) were isolated from marine derived *P. lilacinus* ZBY-1 [46,47].

A novel pyridone alkaloid, militarinone C (**149**), was obtained from the mycelium of *P. militaris* [49]. Six secondary metabolites were obtained from *P. tenuipes*, which include (4*S*,10*R*)-4-hydroxy-8-oxygen-10-methyl solactone (**150**), tenuipesine A (**151**), paecilomycines B, C (**152, 153**), cepharosporolide C (**154**) (Figure 3) and E (**155**) [50,80,87]. Paecilocin A (**156**) and D (**157**), 4-(2-hydroxyethyl) phenol (**158**), stigmasta 4,6,8(14),22-tetraen-3-one, β-sitosterol (**159**) and stigmasterol (**160**) were reported from *P. variotii* [23,25]. Two bicyclic fatty acids, paecilonic acid A (**161**) and B (**162**), together with two benzannulated spiroketal derivatives, paeciloketal B (**163**) and 1-epi-paeciloketal B (**164**) were obtained from jellyfish-derived strain of *P. variotii* J08NF-1 [27,88]. The metabolites 5-methylresorcinol (**165**) and 2,4-dihydroxy-3,6-dimethylbenzaldehyde (**166**) were isolated from cultures of *P. verticillatus* [68].

Two novel unique spiro[chroman-2,1′(3′H)-isobenzofuran] derivative (**167**), (3R*,5*E*, 7*E*,9R*,11*E*,13*Z*)-1-((3′aS*,6′aR*)-2-amino-5-oxo-3′a,5′,6′,6′α-tetrahydrofu-ro[3′,2-b]furan-3-yl)-3,7,9,11-tetramethylheptadeca-5,7,11,13-tetraene-1,2-dione (**168**), together with cholesteryl linoleate (**169**), and 2,5-furandimethanol (**170**) were isolated from marine-derived strains of *Paecilomyces* [3,89].

A diterpenoid, paecilomycine B (**171**), with a five-membered lactone ring, and three labdane diterpenoids, botryosphaerin E (**172**), agathic acid (**173**), and *rel*-(1*R*,3*S*,4a*S*,5*R*,8a*S*)-5-[(3*E*)-4- carboxy-3-methylbut-3-en-1-yl]decahydro-3-hydrxy-1,4a-dimethyl-6-methylidenenaphthalene-1-carboxylic acid (**174**) were identified from the solid culture of *Paecilomyces* sp. ACCC 37762 [90]. A number of β-resorcylic acid lactones paecilomycin A–D (**175**–**178**), paecilomycin G–L (**179**–**184**), paecilomycin N, O (**185**,**186**), 4′-hydroxymonocillin IV (**187**), 4′-methoxymonocillin IV (**188)**, zeaenol (**189**), aigialospirol (**190**), zearalenone (**191**), 7′-dehydrozearalenone (**192**), *trans*-7′,8′- dehydrozearalenol (**193**), monocillin I (**194**), monocillin Ⅲ (**195**), radicicol (**196**), lasicicol (**197**), and hypothemycin (**198**) were produced by a strain of *Paecilomyces* sp. SC0924 [30,31,32,78]. Furthermore, the compound 7′-dehydrozearalenone (**192**) was firstly isolated from *Gibberella zeae* [91].

The metabolites 1,5-dideoxy-3-C-methyl-arabitol (**199**) and adenosine (**200**) were identified from a strain of *Paecilomyces* sp. CAFT156 [38]. Several compounds, including a indolinepeptide, 3β,5-dihydroxy-l-*N*-methyl-indoline-2β-carbonyl amino-d-alanyl-erythro-β-hydoxyisoleucinyl-glycine (**201**), (4*E*, 8*E*, 2*S*, 2′*R*, 3*R*)-*N*-2′-hydroxy-hexadecanoyl-l-*O*-β-d-glucopyranosyl-9-methyl-4, 8-sphingadienin (**202**), alloxazine (**203**), along with the ergosterol derivatives, 3β,5α-dihydroxy-ergosta-7,22-diene (**204**), 5α,6α-epoxy-(22*E*,24*R*)-ergosta-8(14),22-diene-3β,7α-diol (**205**), were isolated from *Paecilomyces* sp. J300 [2,55]. Two cadinane-type sesquiterpenoids, paecilacadinol A and B (**206, 207**), two drimane-type sesquiterpenoids, ustusol D (**208**) and ustusol E (**209**), and the four analogs, deoxyuvidin B (**210**), 3β,9α,11-trihydroxy-6-oxodrim-7-ene (**211**), 2α,11-dihydroxy-6-ox-odrim-7-ene (**212**), and ustusol B (**213**) were obtained from the endophytic fungus *Paecilomyces* sp. TE-540 [70]. The metabolite, paecilin B (**214**) [57], and nine cyclic peptides, viscumamide (**215**), cyclo(Pro-Iso) (**216**), cyclo(Phe-Gly) (**217**), cyclo(Phe-Ana) (**218**), cyclo(Gly-Pro) (**219**), cyclo(Gly-Leu) (**220**), cyclo(Trp-Ana) (**221**), necoeshinulin A (**222**), and cyclo(Pro-Thr) (**223**) were identified from *Paecilomyces* sp. (tree 1–7) [92].

## 3. Conclusions

Since *Paecilomyces* were first described, many have been proven to be insect pathogens. As a result of the hardiness, wide adaptability, and ease of culture of most species of *Paecilomyces*, they play an important role in pest control, medicine, functional foods, environmental pollution control, and genetic engineering. Furthermore, *Paecilomyces* species are a source of bioactive natural products. At present, more than two hundred metabolites have been isolated and identified from *Paecilomyces*. In this paper, 223 metabolites produced from 13 species and various unidentified species of *Paecilomyces* were reviewed.

The structures of metabolites from *Paecilomyces* vary and have been reported ranging from polyketide, terpenoid, peptide, alkaloid, quinone, pyrone, sterol, fatty acid, xanthone, macrocyclic, pyrenocine analog, to radicicol-type forms. The representative secondary metabolites are the highly toxic linear peptides known as leucinostatins, the tyrosine kinase inhibitors paeciloquinones, the tetramic acid derivative, paecilosetin, and a series of trichothecanes. These metabolites have diverse biological activities, such as antimicrobial, antiviral, antitumor, herbicidal, insecticidal, antiplasmodial, antitrypanosomal, nematicidal, cytotoxic, enzyme inhibitors, phytotoxicity, and radical scavenging. The control effect of *Paecilomyces* is mainly the result of insecticidal activity of its metabolites. Many *Paecilomyces* metabolites not only directly cause disease in insects, but also have indirect insecticidal effect. For example, the fermentation filtrate of *P. lilacinus* showed obvious avoidance of soybean cyst nematode larvae and noticeably inhibited the infection of nematodes in roots [93].

In summary, *Paecilomyces* is a type of fungi with huge potential for development in various applications. With further study, *Paecilomyces* will play an increasingly important role in biological control, medicine and environmental protection.

## Figures and Tables

**Table 1:** The active metabolites derived from *Paecilomyces*.

Metabolites	*Paecilomyces* Strain	Biological Activities	References
terezine D (**1**)	*P. cinnamomeus* BCC 9616	antifungal	[4]
farinomalein (**2**)	*P. farinosus* HF599	antifungal	[6]
phomaligol A (**3**)	*P. lilacinus*	antibacterial	[7,8]
leucinostatin A (**4**)	*P. lilacinus* A-267	antimicrobial, antitumor, uncoupling effect on rat liver mitochondrial function	[10,11,12]
leucinostatin B (**5**)	*P. lilacinus* A-267	antimicrobial, antitumor, uncoupling effect on rat liver mitochondrial function	[10,11,12]
phomapyrone C (**6**)	*P. lilacinus*	antibacterial	[7,13]
leucinostatin D (**7**)	*P. marquandii*	antimicrobial, cytotoxic, phytotoxicity	[15]
leucinostatin H (**8**)	*P. marquandii*	antimicrobial	[16]
leucinostatin K (**9**)	*P. marquandii*	antimicrobial	[16]
sorbicillinoid (**10**)	*P. marquandii*	antibacterial	[17]
beauvericin (**11**)	*P. tenuipes* BCC 1614	antimicrobial, cytotoxic, insecticidal	[18]
beauvericin A (**12**)	*P. tenuipes* BCC 1614	antimicrobial, cytotoxic, insecticidal	[18]
paecilocin B (**13**)	*P. variotii*	antibacterial	[23]
paecilocin C (**14**)	*P. variotii*	antibacterial	[23]
semi-viriditoxin (**15**)	*P. varioti*	antibacterial	[24]
semi-viriditoxic acid (**16**)	*P. varioti*	antibacterial	[24]
lawsozaheer (**17**)	*P. varioti*	antibacterial	[25]
varioloid A (**18**)	*P. variotii* EN-291	antifungal	[26]
varioloid B (**19**)	*P. variotii* EN-291	Antifungal, cytotoxic	[26]
paeciloketal A (**20**)	*P. variotii* J08NF-1	antibacterial	[27]
paecilospirone (**21**)	*Paecilomyces* sp.	antibacterial	[28]
paeciloxocin A (**22**)	*Paecilomyces* sp.	antifungal, cytotoxic	[29]
paecilomycin M (**23**)	*Paecilomyces* sp. SC0924	antifungal	[31]
monocillin VI (**24**)	*Paecilomyces* sp. SC0924	antifungal	[32]
monocillin VII (**25**)	*Paecilomyces* sp. SC0924	antifungal	[32]
aigilomycin B (**26**)	*Paecilomyces* sp. SC0924	antifungal	[30]
aigilomycin C (**27**)	*Paecilomyces* sp. SC0924	antifungal	[30]
aigilomycin D (**28**)	*Paecilomyces* sp. SC0924	antifungal	[30]
1′,2′-epoxy aigialomycin D (**29**)	*Paecilomyces* sp. SC0924	antifungal	[30]
LL-Z1640-1 (**30**)	*Paecilomyces* sp. SC0924	antifungal	[30]
monocillin II (**31**)	*Paecilomyces* sp. SC0924	antibacterial	[33]
monocillin IV (**32**)	*Paecilomyces* sp. SC0924	antibacterial	[33]
monorden D (**33**)	*Paecilomyces* sp. SC0924	antibacterial	[33]
paeciloside A (**34**)	*Paecilomyces* sp. CAFT156	antibacterial, cytotoxic	[38]
acremoauxin A (**35**)	*Paecilomyces* sp. CAFT156	antibacterial, cytotoxic	[38]
paeciloxanthone (**36**)	*Paecilomyces* sp. (tree 1–7)	antimicrobial, cytotoxic, enzyme inhibition	[39]
paecilodepsipeptide A (**37**)	*P. cinnamomeus* BCC 9616	cytotoxic, enzyme inhibition, antimalarial	[40]
farinosone A (**38**)	*P. farinosus* RCEF 0101	induce neurite outgrowth in the PC-12 cell line	[41]
farinosone B (**39**)	*P. farinosus* RCEF 0101	cytotoxic	[41,42]
farinosone C (**40**)	*P. farinosus* RCEF 0101	induce neurite outgrowth in the PC-12 cell line	[41]
paecilosetin (**41**)	*P. farinosus*	cytotoxic	[42]
(+)-*N*-deoxymilitarinone A (**42**)	*P. farinosus* RCEF 0097	cytotoxic, induce neurite sprouting in PC-12 cell line	[43]
(3S,6S)-3,6-dibenzylpiperazine-2,5-dione (**43**)	*P. formous* 17D47-2	cytotoxic	[44]
gunnilactam A (**44**)	*P. gunnii*	cytotoxic	[45]
1,2-dilinolylglycero-*O*-4′-(*N*,*N*,*N*-trimethyl) homoserine (**45**)	*P. lilacinus* ZBY-1	cytotoxic	[46]
methyl myristate (**46**)	*P. lilacinus* ZBY-1	cytotoxic	[46]
cerebroside B (**47**)	*P. lilacinus* ZBY-1	cytotoxic	[47]
cerebroside C (**48**)	*P. lilacinus* ZBY-1	cytotoxic	[47]
cerebroside D (**49**)	*P. lilacinus* ZBY-1	cytotoxic	[47]
militarinone A (**50**)	*P. militaris*	neurotrophic effect in PC-12 cells	[49]
militarinone B (**51**)	*P. militaris* *P. farinosus* RCEF 0097	cytotoxic	[43,48]
militarinone D (**52**)	*P. militaris* *P. farinosus* RCEF 0097	cytotoxic	[43,48]
(3*S*)-6-phenethyl-3-isopropyl-1-methyl-2,5-diketopiperazine (**53**)	*P. tenuipes*	cytotoxic	[50]
indolyl-6,10b-dihydro-5a*H*-[1]benzofuro[2,3-*b*]indole derivative (**54**)	*P. variotii* EN-291	cytotoxic	[51]
dihydrocarneamide A (**55**)	*P. variotii* EN-291	cytotoxic	[52]
*iso*-notoamide B (**56**)	*P. variotii* EN-291	cytotoxic	[52]
UCE1022 (**57**)	*Paecilomyces* sp.	cytotoxic	[53]
saintopin (**58**)	*Paecilomyces* sp.	cytotoxic	[54]
paecilomycin P (**59**)	*Paecilomyces* sp. SC0924	cytotoxic	[32]
monocillin VI (**60**)	*Paecilomyces* sp. SC0924	cytotoxic	[32]
monocillin VII (**61**)	*Paecilomyces* sp. SC0924	cytotoxic	[32]
5α,6α-epoxy-(22*E*,24*R*)-ergosta-8,22-diene-3β,7α-diol (**62**)	*Paecilomyces* sp. J300	cytotoxic	[55]
ergosta-4,6,8(14),22-tetraene-3-one (**63**)	*Paecilomyces* sp. J300	cytotoxic	[55]
3β,5α-dihydroxy-6β-methoxyer-gosta-7,22-diene (**64**)	*Paecilomyces* sp. J300	cytotoxic	[55]
ergosterol (**65**)	*Paecilomyces* sp. J300	cytotoxic	[55]
ergosterol endoperoxide (**66**)	*Paecilomyces* sp. J300	cytotoxic	[55]
paecilin A (**67**)	*Paecilomyces* sp. (tree 1–7)	cytotoxic	[56]
secalonic acid D (**68**)	*Paecilomyces* sp. (tree 1–7)	cytotoxic, enzyme inhibition	[57]
secalonic acid A (**69**)	*Paecilomyces* sp. (tree 1–7)	cytotoxic	[58]
tenellic acid A (**70**)	*Paecilomyces* sp. (tree 1–7)	cytotoxic	[58]
tetracenomycin D (**71**)	*Paecilomyces* sp. (tree 1–7)	cytotoxic	[59]
physcioin (**72**)	*Paecilomyces* sp. (tree 1–7)	cytotoxic	[59]
emodin (**73**)	*Paecilomyces* sp. (tree 1–7)	cytotoxic	[59]
chrysophanol (**74**)	*Paecilomyces* sp. (tree 1–7)	cytotoxic	[59]
1,4-dihydroxy-2-methy anthraquinone (**75**)	*Paecilomyces* sp. (tree 1–7)	cytotoxic	[59]
paecilopeptin (**76**)	*P. carneus*	inhibiting human cathepsin S	[60]
paeciloquinone A (**77**)	*P. carneus* P-177	enzyme inhibition	[61]
paeciloquinone C (**78**)	*P. carneus* P-177	enzyme inhibition	[61]
paeciloquinone D (**79**)	*P. carneus* P-177	enzyme inhibition	[63]
YW3548 (**80**)	*P. formosus* LHL10	enzyme inhibition	[64]
paecilomycone A (**81**)	*P. gunnii*	enzyme inhibition	[65]
paecilomycone B (**82**)	*P. gunnii*	enzyme inhibition	[65]
paecilomycone C (**83**)	*P. gunnii*	enzyme inhibition	[65]
paecilomide (**84**)	*P. lilacinus*	enzyme inhibition	[66]
Sphingofungin E (**85**)	*P. variotii* ATCC 74097	enzyme inhibition	[67]
Sphingofungin F (**86**)	*P. variotii* ATCC 74097	enzyme inhibition	[67]
verticilatin (**87**)	*P. verticillatus*	enzyme inhibition	[68]
3α-hydroxy-3,5-dihydro ML-236C (**88**)	*P. viridis* L-68	enzyme inhibition	[69]
12-hydroxyalbrassitriol (**89**)	*Paecilomyces* sp. TE-540	enzyme inhibition	[70]
2-hydroxyalbrassitriol (**90**)	*Paecilomyces* sp. TE-540	enzyme inhibition	[70]
phenopicolinic acid (**91**)	*Paecilomyces* sp. AF2562	enzyme inhibition	[71]
kurasoin A (**92**)	*Paecilomyces* sp. FO-3684	enzyme inhibition	[72]
kurasoin B (**93**)	*Paecilomyces* sp. FO-3684	enzyme inhibition	[72]
catenioblin C (**94**)	*P. cateniobliquus* YMF1.01799	promoted the growth of the larvae of cotton bollworm	[73]
phomalactone (**95**)	*P. cateniobliquus* YMF1.01799	inhibition cotton bollworm	[73]
cerebrosides A (**96**)	*P. lilacinus* ZBY-1	nematicidal	[74]
4-(4′-carboxy-2′-ethyl-hydroxypentyl)-5,6-dihydro-6-methyl-cyclobut[b]pyridine-3,6-dicarboxylic acid (**97**)	*Paecilomyces* sp. YMF1.01761	nematicidal	[75]
paeciloxazine (**98**)	*Paecilomyces* sp. BAUA3058	Nematicidal, insecticidal	[76]
harzialactone A (**99**)	*Paecilomyces* sp. 7A22	insecticidal	[77]
paecilomycin E (**100**)	*Paecilomyces* sp. SC0924	antiplasmodial	[78]
paecilomycin F (**101**)	*Paecilomyces* sp. SC0924	antiplasmodial	[78]
aigilomycin B (**102**)	*Paecilomyces* sp. SC0924	antiplasmodial	[78]
aigialomycin F (**103**)	*Paecilomyces* sp. SC0924	antiplasmodial	[78]
pyrenocine I (**104**)	*Paecilomyces* sp. FKI-3573	antitrypanosomal	[79]
*pyrenocine A* (**105**)	*Paecilomyces* sp. *FKI-3573*	antitrypanosomal	[79]
pyrenocine B (**106**)	*Paecilomyces* sp. FKI-3573	antitrypanosomal	[79]
citreoviridin (**107**)	*Paecilomyces* sp. FKI-3573	antitrypanosomal	[79]
spirotenuipesine A (**108**)	*P. tenuipes*	activity in neurotrophic factor biosynthesis in glial cells	[81]
spirotenuipesine B (**109**)	*P. tenuipes*	activity in neurotrophic factor biosynthesis in glial cells	[81]
paecilomycine A (**110**)	*P. tenuipes*	activity in neurotrophic factor biosynthesis in glial cells	[80]
formoxazine (**111**)	*P. formosus*	radical-scavenging activity	[82]
terreusinone (**112**)	*P. formosus*	UV-A absorbing activity	[83]
3-[(2Z)-1-oxo-2-buten-1-yl]oxazolidin-2-one (**113**)	*P. formosus*	radical-scavenging activity	[82]
14-hydroxycornexistin (**114**)	*P. variotii*	herbicidal	[84]
cornexistin (**115**)	*P. variotii* SANK 21086	herbicidal	[85,86]
